# Dengue Incidence Trends and Its Burden in Major Endemic Regions from 1990 to 2019

**DOI:** 10.3390/tropicalmed7080180

**Published:** 2022-08-12

**Authors:** Na Tian, Jin-Xin Zheng, Zhao-Yu Guo, Lan-Hua Li, Shang Xia, Shan Lv, Xiao-Nong Zhou

**Affiliations:** 1 National Institute of Parasitic Diseases, Chinese Center for Disease Control and Prevention (Chinese Center for Tropical Diseases Research), Shanghai 200025, China; 2 School of Public Health, Weifang Medical University, Weifang 261000, China; 3 NHC Key Laboratory of Parasite and Vector Biology, Shanghai 200025, China; 4 WHO Collaborating Centre for Tropical Diseases, Shanghai 200025, China; 5 National Center for International Research on Tropical Diseases, Shanghai 200025, China; 6 School of Global Health, Chinese Center for Tropical Diseases Research, Shanghai Jiao Tong University School of Medicine, Shanghai 200025, China

**Keywords:** dengue, incidence, burden, endemic regions, model

## Abstract

Background: Dengue has become one of the major vector-borne diseases, which has been an important public health concern. We aimed to estimate the disease burden of dengue in major endemic regions from 1990 to 2019, and explore the impact pattern of the socioeconomic factors on the burden of dengue based on the global burden of diseases, injuries, and risk factors study 2019 (GBD 2019). Methods: Using the analytical strategies and data from the GBD 2019, we described the incidence and disability-adjusted life years (DALYs) of dengue in major endemic regions from 1990 to 2019. Furthermore, we estimated the correlation between dengue burden and socioeconomic factors, and then established an autoregressive integrated moving average (ARIMA) model to predict the epidemic trends of dengue in endemic regions. All estimates were proposed as numbers and age-standardized rates (ASR) per 100,000 population, with uncertainty intervals (UIs). The ASRs of dengue incidence were compared geographically and five regions were stratified by a sociodemographic index (SDI). Results: A significant rise was observed on a global scale between 1990 and 2019, with the overall age-standardized rate (ASR) increasing from 557.15 (95% UI 243.32–1212.53) per 100,000 in 1990 to 740.4 (95% UI 478.2–1323.1) per 100,000 in 2019. In 2019, the Oceania region had the highest age-standardized incidence rates per 100,000 population (3173.48 (95% UI 762.33–6161.18)), followed by the South Asia region (1740.79 (95% UI 660.93–4287.12)), and then the Southeast Asia region (1153.57 (95% UI 1049.49–1281.59)). In Oceania, South Asia, and Southeast Asia, increase trends were found in the burden of dengue fever measured by ASRs of DALY which were consistent with ASRs of dengue incidence at the national level. Most of the countries with the heaviest burden of dengue fever occurred in areas with low and medium SDI regions. However, the burden in high-middle and high-SDI countries is relatively low, especially the Solomon Islands and Tonga in Oceania, the Maldives in South Asia and Indonesia in Southeast Asia. The age distribution results of the incidence rate and disease burden of dengue fever of major endemic regions showed that the higher risk and disease burden are mainly concentrated in people under 14 or over 70 years old. The prediction by ARIMA showed that the risk of dengue fever in South and Southeast Asia is on the rise, and further prevention and control is warranted. Conclusions: In view of the rapid population growth and urbanization in many dengue-endemic countries, our research results are of great significance for presenting the future trend in dengue fever. It is recommended to policy makers that specific attention needs to be paid to the negative impact of urbanization on dengue incidence and allocate more resources to the low-SDI areas and people under 14 or over 70 years old to reduce the burden of dengue fever.

## 1. Introduction

This study used the analytical strategies and data from the GBD 2019 to describe the incidence and disability-adjusted life years (DALYs) of dengue in major endemic regions from 1990 to 2019, then estimated the correlation between dengue burden and socioeconomic factors, and established an autoregressive integrated moving average (ARIMA) model to predict the epidemic trends in dengue. We found that within a global scale, our model shows an increase in the incident cases of dengue between 1990 and 2019; the upward trends in ASR occurred in middle-, high-middle-, and high-SDI areas and mostly geographic regions, except the Caribbean, Central Sub-Saharan Africa, Western Sub-Saharan Africa, Southern Sub-Saharan Africa, and Eastern Sub-Saharan Africa, and the downward trends in ASR also showed in low- and low-middle-SDI areas. The age-standardized incidence rates of dengue per 100,000 population are reported to be the highest in Tonga and Niue, followed by Palau and Fiji in the Oceania region. In South Asia, the age-standardized incidence rate for dengue was highest in India, but followed by Sri Lanka, Bangladesh and Nepal. In Southeast Asia; the top four countries with an age-standardized incidence of dengue fever are the Philippines, Malaysia, Lao People’s Democratic Republic, and Cambodia. In Oceania, South Asia, and Southeast Asia, there are increase trends in the age-standardized DALY rate which are consistent with the age-standardized incidence rate at the national level. Most of the countries with the heaviest burden of dengue fever are areas with low and medium SDI levels. However, the burden of dengue fever in high-middle- and high-SDI countries is relatively low, especially the Solomon Islands and Tonga in Oceania, the Maldives in South Asia and Indonesia in Southeast Asia. The age distribution results of the incidence rate and disease burden of dengue fever of major endemic regions showed that the higher risk and disease burden are mainly concentrated in people under 14 or over 70 years old. ARIMA predicts the risk of dengue fever in South and Southeast Asia is on the rise, and further prevention and control are needed. Policy makers should now pay more attention to the negative impact of urbanization on dengue fever and allocate more resources to low-SDI areas and people under 14 or over 70 years old to reduce the burden of dengue fever.

Today, dengue fever, caused by the dengue virus (DENV) transmitted by *Aedes aegypti* or *Aedes albopictus* [1], is one of the most widespread and rapidly spreading mosquito-borne infectious diseases in humans [2]. DENV belongs to the genus *Flavivirus* of the family *Flaviridae* with four different serotypes (DENV-1, DENV-2, DENV-3 and DENV-4) [3]. The patients infected with any of the serotypes may present as dengue fever, dengue hemorrhagic fever, or dengue shock syndrome. Dengue is mainly distributed in tropical and subtropical areas of Africa, Southeast Asia, the Pacific Ocean, the Western Mediterranean and the Americas [4].

According to a report by the World Health Organization (WHO), dengue fever is currently endemic in 128 countries; 3.6 billion people live in areas at risk of disease transmission, with an estimated 390 million infected each year and 96 million cases showing some degree of clinical symptoms [5]. Dengue has caused a huge economic and disease burden in endemic countries [6]; about 70% of the global burden of dengue disease occurs in Asia [7]. As a result, the floating population is constantly increasing, and the national dengue control program is becoming increasingly grim.

Currently, no licensed vaccines or specific effective antiviral therapies have been used in the dengue program [8]. Although Dengvaxia is the only dengue vaccine approved by the FDA and has been licensed in 20 countries, the WHO does not recommend its use in seronegative patients [9]. Due to the lack of antiviral drugs and safe and effective vaccines, the only available preventive measure is vector control, which is mostly dependent on community participation [10]. To strengthen vector control, the WHO established the global vector control response 2017–2030 (GVCR) [11], which initiated cooperation within and outside the health sector to strengthen community participation, expand and integrate vector control tools and methods, and improve monitoring and evaluation. More countries have adjusted their national control programs according to GVCR [12], but at present, good examples of the GVCR principles are still limited. For example, while intensive vector control efforts have successfully suppressed Aedes mosquito populations, from an Aedes housing index of more than 50% in the 1960s to 1–2% now, Singapore remains prone to dengue outbreaks [13,14,15]. Increased population density and low herd immunity due to sustained low dengue transmission are factors that may contribute to the resurgence of dengue fever in Singapore [16,17]. In spite of the vector control of dengue achieving remarkable results, the global status of dengue control programs is still facing increasing problems.

Dengue imposes a heavy financial burden on both the government and individuals. Dengue costs an average of USD 2.1 billion a year in the Americas, not including vector control, more than other viral diseases [18]. In Southeast Asia, an estimated 2.9 million dengue outbreaks and 5906 deaths occur each year, with an annual economic burden of USD 950 million [19]. Its rapid rise in the global burden of dengue has been related to demographic and social changes over the past 50 to 60 years, including unprecedented population growth, climate change, uncontrolled urbanization, increased mobility, collapse of public health infrastructure as well as vector control. Understanding the burden of dengue fever is essential for the rational allocation of limited resources. The disease burden of dengue fever should not be underestimated and is full of challenges for health policy makers. Assessing the epidemic risk and disease burden of dengue fever will help cope with the growing global threat.

Recently, the dengue burden by using the global burden of diseases, injuries, and risk factors study has been reported [20,21]. However, there is still a lack of important information related to dengue transmission, e.g., the linkages between the burden of dengue fever and the social demographic status at national level, and the transmission trend of dengue fever in major endemic regions. Therefore, we assessed the dengue transmission patterns in major endemic regions by establishing an autoregressive integrated moving average model (ARIMA), after exploring the relationship between the age-standardized rate (ASR) of dengue incidence and its disability-adjusted life years (DALYs) weighted by sociodemographic index (SDI) from 1990 to 2019 based on the GBD data in 2019.

## 2. Methods

### 2.1. Ethics Statement

All data were obtained from the GBD database. The GBD study was approved by the ethics board of the University of Washington.

### 2.2. Data Collection

Data were derived from Global Health Data Exchange which is a publicly available source (http://ghdx.healthdata.org/gbd-results-tool) (accessed on 4 February 2021). We employed data from the GBD 2019 conducted by the Institute of Health Metrics and Evaluation (IHME) including information on dengue fever from 1990 to 2019 in 204 countries and regions. The GBD study has estimated the incidence, prevalence and YLDs of 369 diseases and injuries, 3484 sequelae (i.e., the disabling consequences of these diseases and injuries), and 87 risk factors based on age, gender and location. A variety of indicators to measure health losses in the population, such as cases, morbidity and disability-adjusted life years were extracted from GBD 2019 for further analysis. Those data were sorted by incident cases and their 95% uncertainty intervals (UI), and DALYs for dengue were calculated for each of the regions and countries from 1990 to 2019. Five regions of the world were stratified according to SDI, e.g., low, low-middle, middle, high-middle, and high regions. A total of 204 countries and regions were included for geographical comparison, which were divided into 45 subcontinental regions, such as Central Asia, Central Europe, Southeast Asia and so on.

### 2.3. Statistical Analysis

Comparison analysis was performed among the number of cases per 100,000 population worldwide and the ASR of dengue incidence was stratified by five SDI regions, 45 subcontinental regions, and sex levels, in order to understand the dengue transmission status in the global endemic areas of tropical islands. ASR, DALYs, and their relationship with SDI were described for each country in the three most prevalent regions using the fit spline models [22]. UI was calculated based on 1000 estimated values of drawing level for each parameter; 95% UI was defined by the 25th and 97.5th values of 1000 estimated values; and 95% UI (excluding 0) was considered to have statistical significance. All statistical analyses in this study were performed using R software.

### 2.4. ARIMA Model

The ARIMA model is a differential integrated moving average autoregressive model, also known as an integrated moving average autoregressive model, which is one of the time series forecasting analysis methods. In ARIMA (p, d, q), AR is “autoregressive” and p is the number of autoregressive terms. MA is the “moving average”, q is the number of terms in the moving average, and d is the number of differences (order) made to make it a stationary sequence [23]. A time series was generated using R statistical software to analyze the trend in dengue cases. We used R software to generate the ASR of dengue fever in three major epidemic regions from 1990 to 2019, determined the time series for predictive analysis, and then applied the ARIMA model to predict the incidence of dengue fever from 2020 to 2030.

## 3. Results

### 3.1. Global Dengue Incidence

**Figure 1:**
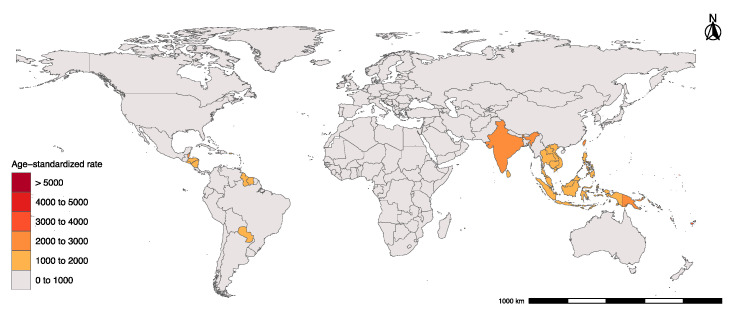
Age-standardized incidence rate of Dengue in countries and regions globally, 2019.

Globally, our model shows an increase in dengue incident cases between 1990 and 2019, from 30.67 million (13.22 million–67.07 million) in 1990, to 56.88 million (37.08 million–101.35 million) in 2019 (Table 1). From 1990 to 2019, the incidence of dengue had a clearly global upward trend, with the total ASR increasing from 557.15 (95% UI 243.32–1212.53) per 100,000 in 1990 to 740.4 (95% UI 478.2–1323.1) per 100,000 in 2019. The rising trends in ASR occurred in middle, high-middle, and high SDI areas and mostly geographic regions, except the Caribbean, Western Sub-Saharan Africa, Eastern Sub-Saharan Africa, Central Sub-Saharan Africa, and Southern Sub-Saharan Africa (Table 1; Figure S1a; Figure S1b). However, the ASR in low and low-middle SDI regions also showed a fluctuation trend, declining from 674.4 (95% UI 312.1–1861.22) per 100,000 in 1990 to 619.51 (95% UI 283.41–1614.6), and 1337.13 (95% UI 383.83–3760.47) per 100,000 in 1990 to 1317.83 (95% UI 554.94–3275.4) in 2019, respectively (Table 1; Figure S1a). In terms of gender, the age-standardized incidence rate per 100,000 for male and female was basically similar from 1990 to 2019, but the incidence rate for female was slightly higher (Table 1; Figure S1c). Dengue is endemic mainly in tropical and subtropical areas. Among all the regions, the most severely prevalent areas of dengue fever are in the regions of Oceania (3173.48 (95% UI 762.33–6161.18)), South Asia (1740.79 (95% UI 660.93–4287.12)) and Southeast Asia (1153.57 (95% UI 1049.49–1281.59)) per 100,000 in 2019 (Table 1; Figure 1; Figure S1b).

### 3.2. Burden of Dengue in Three Major Endemic Regions

Among 21 geographical regions in 1990, the South Asia region had the highest age-standardized incidence rates per 100,000 population (1663.51 [95% UI 444.97–4250.09]), followed by the Southeast Asia region (1036.23 [95% UI 600.63–2682.51]), and then the Oceania region (1009.45 [95% UI 793.94–1198.93]). Additionally, in 2019, the Oceania region had the highest age-standardized incidence rates per 100,000 population (3173.48 [95% UI 762.33–6161.18]), followed by the South Asia region (1740.79 [95% UI 660.93–4287.12]), and then the Southeast Asia region (1153.57 [95% UI 1049.49–1281.59]). Region-specific results are included in Table 1. In view of the above results, we describe the disease burden of dengue in major endemic regions, represented by Oceania, South and Southeast Asia, through the specific metrics as DALYs.

### 3.3. Oceania Dengue Burden

**Figure 2:**
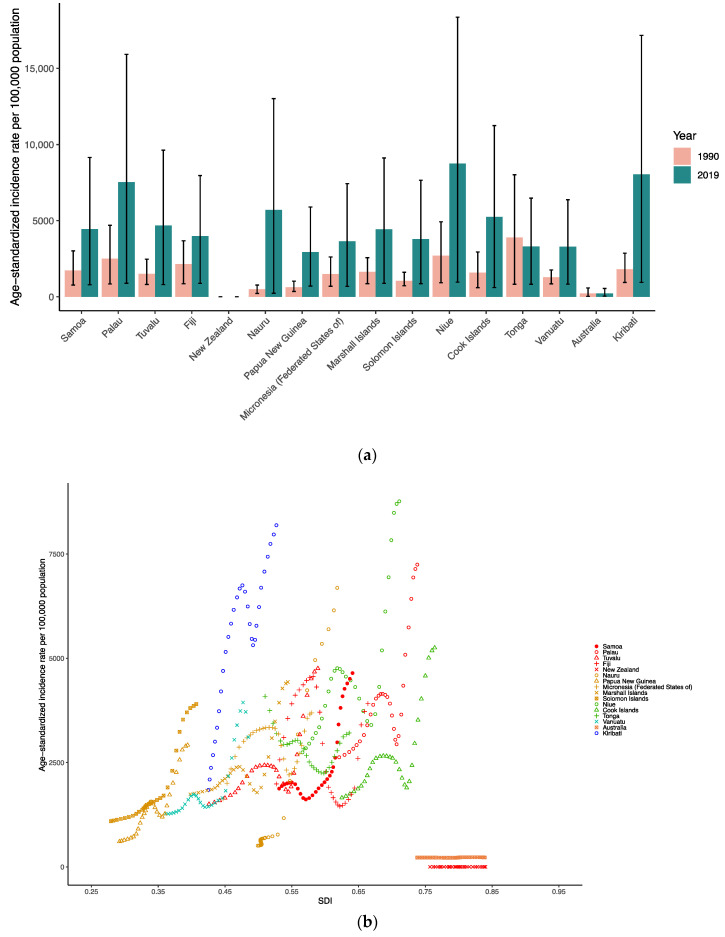
(**a**). Age-standardized incidence rate per 100,000 people with dengue of Oceania countries in 1990 and 2019. (**b**). Age-standardized incidence rate of dengue for Oceania countries by SDI, 1990–2019.

At the national level in the Oceania region in 1990, the age-standardized incidence rate per 100,000 population of dengue was highest in Tonga (3905.13 [821.13–8019.19]), followed by Niue (2707.12 [920.78–4924.34]), Palau (2510.37 [842.14–4696.64]) and Fiji (2158.6 [852.49–3676.16]). By contrast, in 2019, the age-standardized incidence rate per 100,000 population of dengue was highest in Niue (8749.54 [958.61–18365.54]), followed by Kiribati (8050.59 [944.28–17167.95]), Palau (7536.07 [888.63–15922.13]) and Nauru (5703.39 [231.39–13013.35]) (Table S1 and Figure 2a). Figure 2b presents the national-level age-standardized incidence rates from 1990 to 2019 and their relationship with the SDI. The results show that the age-standardized incidence rate reaches its peak when the SDI is 0.5 to 0.75, and then decreases as the SDI value increases. The age-standardized incidence rates of Niue, Kiribati and Palau from 1990 to 2019 were much higher than expected.

**Figure 3:**
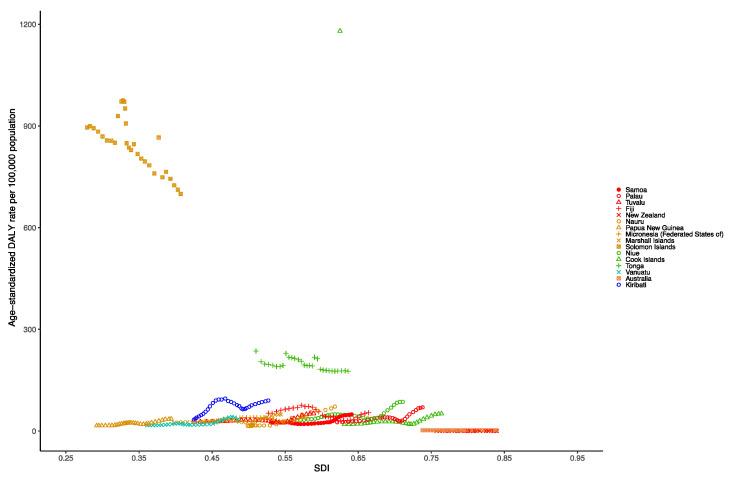
Age-standardized DALY rate of dengue for Oceania countries by SDI, 1990–2019.

At the national level in the Oceania region in 1990, the age-standardized DALY rate per 100,000 population of dengue was highest in the Cook Islands (1475.96 [1343.00–1630.60]), followed by the Solomon Islands (736.51 [400.88–1285.77]), Tonga (231.16 [127.71–378.25]) and Fiji (53.17 [28.98–88.27]). Nevertheless, in 2019, the age-standardized DALY rate per 100,000 population of dengue was highest in the Solomon Islands (643.73 [412.16–1325.22]), followed by Tonga (178.12 [101.98–308.74]), Kiribati (88.04 [8.52–275.07]) and Niue (86.70 [9.32–230.66]) (Table S2 and Figure S2). Figure 3 shows the national-level age-standardized DALY rates from 1990 to 2019 and their relationship with the SDI. The results show that the age-standardized DALY rates reach their peak when the SDI is 0.5 to 0.75, and then decrease as the SDI value increases. The age-standardized DALY rates of the Solomon Islands and Tonga from 1990 to 2019 were much higher than expected.

### 3.4. South Asia Dengue Burden

**Figure 4:**
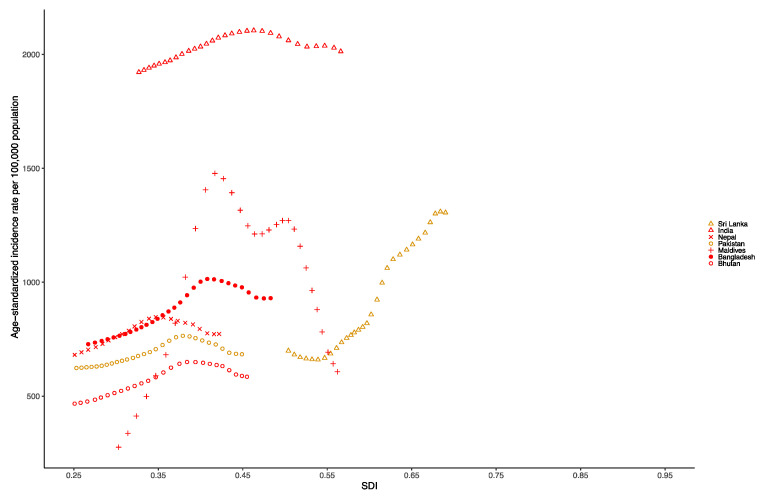
Age-standardized incidence rate of dengue for South Asia countries by SDI, 1990–2019.

At the national level in the South Asia region in 1990, the age-standardized incidence rate per 100,000 population of dengue was highest in India (1943.39 [453.69–5181.79]), followed by Bangladesh (734.57 [396.90–1235.26]), Sri Lanka (696.81 [247.67–1370.52]) and Nepal (639.36 [318.77–1075.22]). Additionally, in 2019, the age-standardized incidence rate per 100,000 population of dengue was still highest in India (2020.83 [667.80–5240.95]), but followed by Sri Lanka (1329.64 [879.91–1821.17]), Bangladesh (922.89 [697.19–1262.38]) and Nepal (764.25 [517.47–1085.56]) (Table S3 and Figure S3). Figure 4 presents the national-level age-standardized incidence rates from 1990 to 2019 and their relationship with the SDI. The results show that the age-standardized incidence rate reaches its peak when the SDI is approximately 0.4 to 0.7, and then decreases as the SDI value increases. The age-standardized incidence rate of India from 1990 to 2019 was much higher than expected.

**Figure 5:**
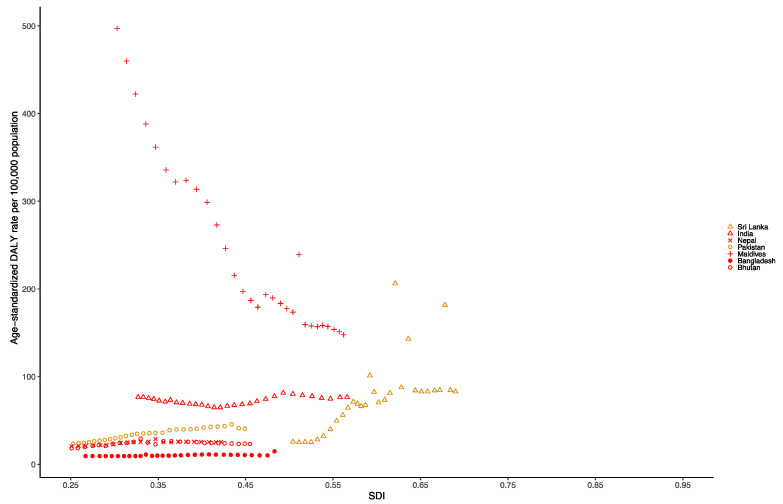
Age-standardized DALY rate of dengue for South Asia countries by SDI, 1990–2019.

At the national level in the South Asia region in 1990, the age-standardized DALY rate per 100,000 population for dengue was highest in Bhutan (345.10 [101.88–692.54]), followed by Nepal (73.33 [16.80–120.49]), Bangladesh (24.46 [13.48–47.47]) and Pakistan (20.25 [6.78–34.14]). Additionally, in 2019, the age-standardized DALY rate per 100,000 population of dengue was still highest in Bhutan (153.32 [69.16–234.50]), followed by Bangladesh (87.78 [24.89–134.18]), Nepal (80.63 [17.47–126.48]) and Pakistan (39.67 [8.44–60.32]) (Table S4 and Figure S4). Figure 5 shows the national-level age-standardized DALY rates from 1990 to 2019 and their relationship with the SDI. The results show that the age-standardized DALY rates reach their peak when the SDI is 0.3 to 0.5, and then decrease as the SDI value increases. The age standardized DALY rates of the Maldives and India as well as Sri Lanka from 1990 to 2019 were much higher than expected.

### 3.5. Southeast Asia Dengue Burden

**Figure 6:**
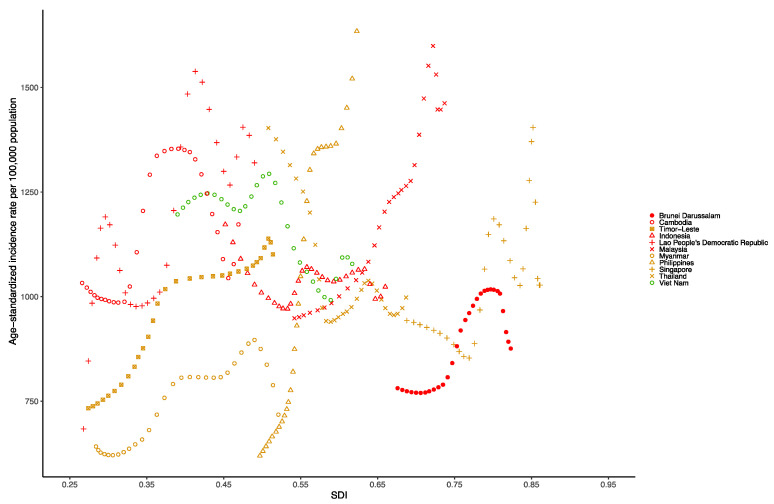
Age-standardized incidence rate of dengue for Southeast Asia countries by SDI, 1990–2019.

At the national level in the Southeast Asia region in 1990, the age-standardized incidence rate per 100,000 population of dengue was highest in Thailand (1389.11 [875.56–1906.71]), followed by Vietnam (1179.1 [870.85–1500.71]), Indonesia (1143.41 [328.46–5182.99]) and Cambodia (1043.82 [758.14–1551.06]). However, in 2019, the age-standardized incidence rate per 100,000 population of dengue was highest in the Philippines (1619.95 [1044.81–2574.39]), followed by Malaysia (1468.76 [894.29–2150.69]), Lao People’s Democratic Republic (1319.73 [796.44–1933.54]) and Cambodia (1171.37 [887.8–-1594.67]) (Table S5 and Figure S5). Figure 6 presents the national-level age-standardized incidence rates from 1990 to 2019 and their relationship with the SDI. The results show that the age-standardized incidence rate reaches its peak when the SDI is 0.4 to 0.8, and then decreases as the SDI value increases. The age-standardized incidence rates of Lao People’s Democratic Republic and Malaysia as well as the Philippines from 1990 to 2019 were much higher than expected.

**Figure 7:**
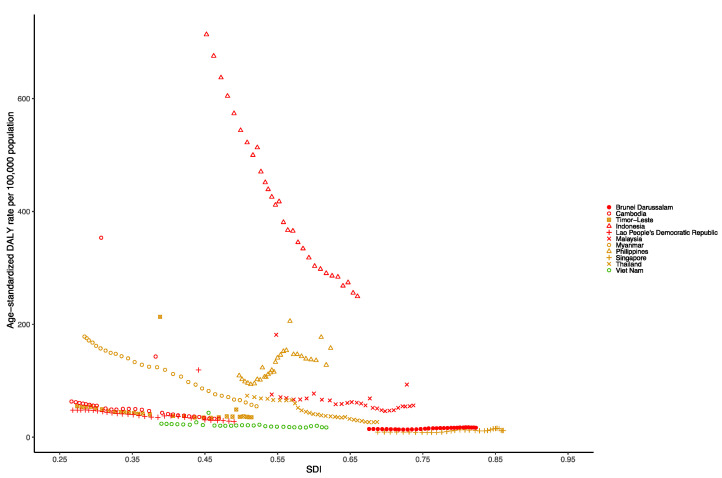
Age-standardized DALY rate of dengue for Southeast Asia countries by SDI, 1990–2019.

At the national level in the Southeast Asia region in 1990, the age-standardized DALY rate per 100,000 population of dengue was highest in Indonesia (600.02 [109.48–998.23]), followed by Myanmar (134.12 [30.52–328.49]), the Philippines (82.73 [30.62–109.37]) and Thailand (72.39 [25.33–119.45]). Additionally, in 2019, the age-standardized DALY rate per 100,000 population for dengue was still highest in Indonesia (290.10 [85.96–397.48]), followed by the Philippines (145.51 [72.15–173.75]), Myanmar (57.88 [22.55–97.73]) and Malaysia (55.95 [35.96–93.76]) (Table S6 and Figure S6). Figure 7 shows the national-level age-standardized DALY rates from 1990 to 2019 and their relationship with the SDI. The results show that the age-standardized DALY rates reach their peak when the SDI is approximately 0.45, and then decrease as the SDI value increases. The age-standardized DALY rate of Indonesia from 1990 to 2019 was much higher than expected.

### 3.6. Age Distribution of Dengue Burden in Three Major Endemic Regions

**Figure 8:**
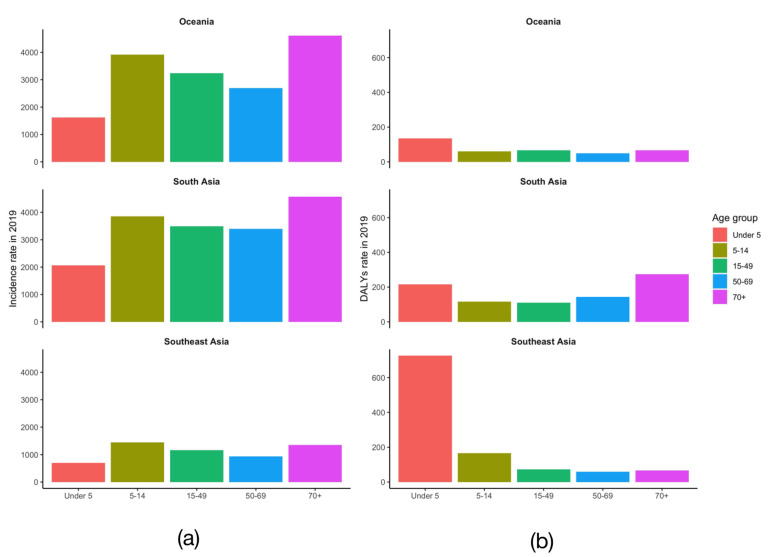
(**a**). Age distribution of dengue incidence in 2019 of Oceania, South Asia and Southeast Asia. (**b**). Age distribution of dengue DALY in 2019 of Oceania, South Asia and Southeast Asia.

Furthermore, we analyzed the age-standardized incidence/DALY rates in five age groups (under 5, 5–14 years, 15–49 years, 50–69 years, over 70 years) of the three major endemic regions in 2019; the results suggest that in Oceania, the highest age-standardized incidence rate per 100,000 population of dengue is in the age group of over 70 years (4610.21 [879.78–10138.58]), followed by the 5–14 years age group (3916.97 [930.97–8969.21]), and the highest age-standardized DALY rate is in the under 5 age group (134.77 [64.16–222.86]), followed by the over 70 years age group (66.78 [23.62–156.11]). In South Asia, the highest ASR is in the over 70 years age group (2284.13 [683.85-5983.69]), followed by the 5–14 years age group (1927.67 [642.01–4783.93]), and the highest DALY is in the under 5 age group (156.11 [21.36–173.13]), followed by the over 70 years age group (137.06 [38.12–193.18]). In Southeast Asia, the highest ASR is the 5–14 years age group (1447.19 [1263.18–1823.01]), followed by the over 70 years age group (1356.32 [1222.49–1538.18]), and the highest DALY is the under 5 age group (726.63 [176.49–1061.41]), followed by the 5–14 years age group (165.86 [66.35–209.75]) (Table S7 and Figure 8).

### 3.7. Prediction of the Incidence Trend of Dengue in Major Endemic Regions-Time Series Model

At present, dengue fever prediction models including time series and machine learning have been developed. In this study, the autoregressive integrated moving average method (ARIMA) was used to predict the incidence trend of dengue fever in major endemic regions.

**Figure 9:**
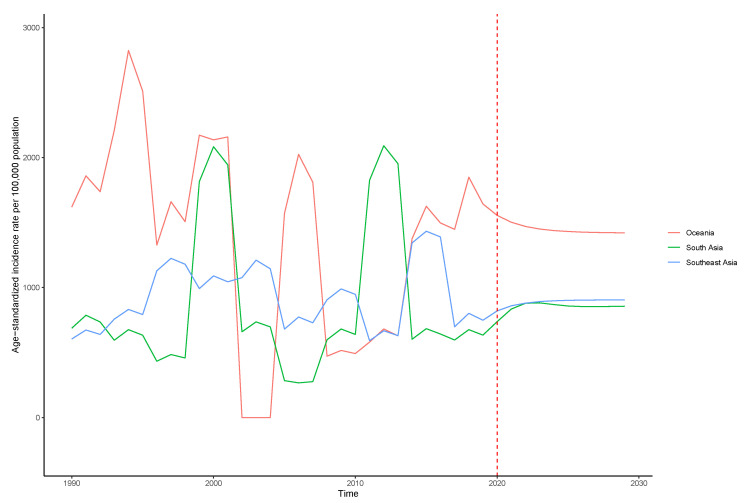
Forecast of dengue incidence rate in Oceania, South Asia, and Southeast Asia from 2020 to 2030.

Figure 9 shows the time series of dengue incidence rate from 1990 to 2019 and the predicted trends from 2020 to 2030 in Oceania, South Asia, and Southeast Asia. In Oceania, the time series shows the outbreak of dengue fever such as the year 1993, 1998, 2006, and 2019. There is a descending trend after 2020 as shown in the ARIMA line. In South Asia, the time series shows the outbreak of dengue fever such as the year 2000 and 2012. There is a rising after 2020 as shown in the ARIMA line. In Southeast Asia, the time series shows the outbreak of dengue fever such as the year 1997, 2002, 2008, and 2015. There is a rising trend after 2020 as shown in the ARIMA line.

## 4. Discussion

In this research, we studied the temporal trends of dengue incidence at the global and regional level, and we made a forecast on the incidence of dengue fever in 204 countries and territories from 1990 to 2019. In general, dengue was increased in both incidence and cases from 1990 to 2019. The dengue incident cases and age-standardized incidence rate (ASR) had increased from 30.7 million and 557.15 per 100,000 in 1990 to 56.9 million and 740.4 per 100,000 in 2019 separately. Our results illustrate the upward trends in dengue incidence in the past three decades globally. However, the time trends of incidence rates vary greatly from region to region and country to country. For example, in low- and low-middle-SDI regions, the ASR of dengue fever has a decreasing trend, which suggests that population growth and aging are the main reasons for the increase in dengue cases in these two regions. Conversely, in middle-, high-middle- and high-SDI regions, the dengue ASR has an increasing trend. Consequently, understanding the prevalence trends of dengue fever is crucial for relevant policy makers to properly allocate medical resources and make targeted interventions.

Beatty Me et al. have shown that dengue fever is mainly prevalent in tropical and subtropical areas, causing a huge economic and disease burden in endemic countries [24]. The GBD 2019 showed that dengue is most prevalent in Oceania, South Asia and as Southeast Asia, which is similar to the GBD 2013 and 2017 findings [20,21]. Our result reports that the age-standardized incidence rate is highest in Oceania, then South Asia, Southeast Asia, tropical Latin America and Central Latin America, which is inconsistent with the results of Stanaway and colleagues [20]. The discrepancies between the results may be due to the increase in data sources and method updates in 2019, as well as climate change and many other influential factors.

Oceania is located in the central and south-central Pacific Ocean in the vast waters north and south of the equator. It is composed of Australia, the smallest continent in the world, and more than 10,000 islands with wide differences in size. The island area accounts for 13.8% of the total area of the continent, most of which lies between latitudes 30° south and north, and belongs to tropical and subtropical regions. South Asia is located between 0° and 37° N, comprising a total of seven countries: Nepal and Bhutan as landlocked countries; India, Bangladesh, and Pakistan as coastal countries; and the Maldives and Sri Lanka as island countries. Most of the region has a tropical monsoon climate. Southeast Asia is located between 10° S and 28°26 N, including the Indo-China Peninsula and Malaysia Archipelago. Scattered over a vast area between the Pacific and Indian Oceans, Malaysia is the world’s largest archipelago, with more than 20,000 islands. The Indo-China Peninsula mostly belongs to the tropical monsoon climate, and the Malay Archipelago mostly belongs to the tropical rainforest climate.

Oceania, South Asia and Southeast Asia all belong to the tropical island area. Tropical island areas are located in the dynamic sensitive zone of sea–land interaction. They have a small area, simple regional structure, high sensitivity and low stability [25]. Most of them are tropical rain forest or tropical monsoon climate, with typical high illumination, high temperature, high humidity and dense grass and other characteristics [26]. This is conducive to the breeding and reproduction of vector insects, and the risk of dengue transmission is increased. At the same time, the island regions are vigorously developing their export-oriented economy and building the “Second Marine Economic Belt”. Tourism, business and trade activities are developing rapidly, the floating population is increasing constantly, and the situation of epidemic prevention and control is becoming increasingly grim. Therefore, this study focused on the disease burden of dengue fever in three major endemic regions, and used mathematical models to predict the incidence trend of dengue fever in endemic areas.

The age-standardized incidence rates of dengue per 100,000 population are estimated to be highest in Tonga and Niue, followed by Palau and Fiji in the Oceania region. In South Asia, the age-standardized incidence rate of dengue was highest in India, but followed by Sri Lanka, Bangladesh, as well as Nepal. In Southeast Asia, the top four countries with an age-standardized incidence of dengue fever are the Philippines, Malaysia, Lao People’s Democratic Republic and Cambodia. Most countries in Oceania showed an upward trend in the age-standardized incidence rate for dengue during 1990–2019, while only Australia and Tonga experienced a descending change at the country level, and New Zealand had no rate reported. Differences in incidence rates among countries in Oceania should not be ignored. It may contribute to the climate of the country as dengue is also a climate-related disease [21]. All countries in South Asia presented an increase in the age-standardized incidence rate from 1990 to 2019. In particular, the growth was most evident in the Maldives, followed by Sri Lanka. This increase may be related to the industrial structure of the Maldives. Its tourism industry is the largest economic pillar, with developed foreign trade and large population flows. It is a tropical-island-type country where dengue fever is prone to epidemics. Among the 11 countries in Southeast Asia, Indonesia, Thailand and Vietnam had a descending trend between 1990 and 2019; the rest of the countries showed an upward trend, especially the Philippines. The Philippines has a monsoon tropical rain forest climate with high temperature and rain, and high humidity. Taking 2019 as an example, the people of the Philippines used various methods to store water during drought in the first half of the year, which also provided an environment for mosquitoes to breed. After the rainy season begins, the ground is prone to standing water, which also provides conditions for the breeding of mosquitoes that spread dengue fever.

In Oceania, we found that the increase in the age-standardized DALY rate was consistent with that of the age-standardized incidence rate at the country levels. The age-standardized DALY rate in the Solomon Islands and the Cook Islands decreased from 1990 to 2019, especially the Cook Islands. Nauru observed an increase in the age-standardized incidence rate during the study period while, in agreement, the age-standardized DALY rate also largely increased. Indeed, this increase is mainly due to strengthened management of dengue fever cases and reduced deaths [27]. In South Asia, we also observed that the increase trend in the age-standardized DALY rate was consistent with that of the age-standardized incidence rate at the country levels, except for Bhutan. Additionally, in Southeast Asia, we observed three countries had an increase trend (Brunei Darussalam, the Philippines and Singapore) which is consistent with that of the incidence rate. At the same time, Thailand and Indonesia showed a significant downward trend, which may be related to the increased attention to dengue prevention and control.

The association between SDI and DALYs in the three major endemic regions at national levels has not been reported by other researchers. The results show that most countries with higher DALYs were with low to middle SDI. Meanwhile, the DALYs were lower in the high-middle- and high-SDI countries except for the Solomon Islands and Tonga in Oceania, the Maldives in South Asia, and Indonesia in Southeast Asia. In countries with low to middle SDI, the greater burden of dengue disease may be due to limited testing tools and medical resources. In most countries with high SDI, the reasons for low DALYs are sturdy disease surveillance, diagnostic system and adequate medical and health resources. Meanwhile, the reasons for countries with high SDI and low DALYs should also consider whether these countries are non-endemic areas or whether they have the primary dengue vector circulating, especially in Australia and New Zealand of the Oceania region [28,29,30]. However, the Solomon Islands and Tonga in Oceania, Maldives in South Asia, and Indonesia in Southeast Asia have a developed economy, flourishing tourism and high urbanization level, which resulted in rapid population growth and, thus, facilitates the spread of dengue fever [16,31,32].

The age distribution results of the incidence rate and disease burden of dengue fever of major endemic areas show that the higher risk and disease burden are mainly concentrated in people under 14 or over 70 years old, which may be related to the lifestyles of young and elderly people, as well as low immunity [33]. Studies have shown that health education can develop good hygiene habits, thereby reducing the risk of dengue transmission [34]. Therefore, health education can be carried out to children through schools and to the elderly through community mobilization to improve the awareness of dengue fever. At the same time, when allocating limited medical resources, we should focus on these groups to reduce the disease burden and economic burden.

At present, dengue fever prediction models including time series and machine learning have been developed. The most commonly used infectious-disease prediction model is the autoregressive integrated moving average method (ARIMA) [35]. In this study, ARIMA was used to predict the incidence trend of dengue fever in the major endemic regions. For Oceania, the time series shows the outbreak of dengue fever such as the years 1993, 1998, 2006, and 2019. There is a descending trend after 2020 as shown in the ARIMA line. For South Asia, the time series shows the outbreak of dengue fever such as the years 2000 and 2012. There is a rising after 2020 as shown in the ARIMA line. For Southeast Asia, the time series shows the outbreak of dengue fever such as the years 1997, 2002, 2008, and 2015. There is a rising trend after 2020 as shown in the ARIMA line. The results of model prediction indicate that the risk of dengue fever in South Asia as well as Southeast Asia is on the rise, and further prevention and control is needed. Urbanization, increased population density, travel and trade activities that allow the virus to spread through transportation have favored the dengue epidemic in South Asia [36]. As no licensed vaccines or specific effective antiviral therapies have been used in the dengue program [8], vector control is still the only effective measure for dengue fever control and prevention [10]. Public health agencies generally combine biotic and abiotic methods for vector control, but biological control methods may disrupt the ecological balance. According to a recently published report on the resistance of dengue vectors to several insecticides endemic to Southeast Asia, insecticide resistance has become a fundamental challenge in many dengue-endemic countries [37]. Timely, valid entomological assessments and appropriate data management provide important information for vector control management, and understanding the mode of action or chemical class of available insecticide products is critical in order to successfully develop and implement resistance management strategies [38]. Other control measures such as eliminating mosquito-breeding grounds and raising public awareness, attitudes and practices towards dengue fever can also be implemented.

ARIMA requires the time series data to be stable, or to be stable after differential processing. Most of the time series data of infectious diseases show nonstationary characteristics, which is a concern for traditional ARIMA models [35]. Another limitation of the ARIMA model is that it cannot create accurate results if the observed data are too small. In Figure 9, the graph is built with 30 observations. Due to the limited data used for prediction, the accuracy of model prediction needs to be further verified.

This study applied data extracted from the GBD database to analyze the incidence trends and the burden of dengue fever for three major endemic regions from 1990 to 2019, and establish a mathematical prediction model. The results of this study can provide a scientific basis for the government to formulate prevention and control measures for dengue fever, especially in tropical island endemic areas, and carry out precise prevention and control for different countries. Model prediction of epidemic trends is an important early warning mechanism in epidemic prevention and control, which is helpful for the forward-looking planning and rational allocation of medical and health resources.

Dengue is a meteorologically related mosquito-borne disease. For this study, natural environment, biology and other factors were not included in the analysis, which may have a certain influence on the results. In the future, we will establish a more systematic and comprehensive model of disease transmission mechanism including pathogen, vector and host, so as to provide a scientific basis for the optimization of dengue prevention and control strategies.

## 5. Conclusions

This study identified dengue epidemic areas and provided a theoretical basis for government and policy makers to allocate health resources reasonably by clarifying the incidence and burden of dengue in major epidemic regions and specific populations. It is recommended to policy makers that specific attention needs to be paid to the negative impact of urbanization on dengue incidence and allocate more resources to the low-SDI areas and people under 14 or over 70 years old to reduce the burden of dengue fever. The prediction of dengue fever epidemic areas in the next 10 years can provide guidance for prevention and control strategies of relevant regions and countries.

## Figures and Tables

**Table 1:** The Incident cases and age-standardized incidence of dengue in 1990 and 2019.

Items	1990		2019	
	Incident Cases	ASR Per 100,000	Incident Cases	ASR Per 100,000
	No. X 10^5^ (95%UI)	No. (95%UI)	No. X 10^5^ (95%UI)	No. (95%UI)
**Overall**	306.7 (132.2–670.7)	557.15 (243.32–1212.53)	568.8 (370.8–1013.5)	740.4 (478.2–1323.1)
**Sex**				
Male	147.3 (63.6–319.3)	532.1 (233.1–1151.8)	274.8 (181.2–482.7)	712.2 (466.5–1256.9)
Femal	159.4 (66.6–347.8)	584.3 (247.8–1273.1)	294 (181.3–529.7)	769.9 (469–1392.9)
**Sociodemographic index**				
Low	34.9 (15.3–96.4)	674.4 (312.1–1861.22)	67.6 (28.8–175.5)	619.51 (283.41–1614.6)
Low-middle	150.3 (41.7–426.1)	1337.13 (383.83–3760.47)	231.1 (98.4–571.5)	1317.83 (554.94–3275.4)
Middle	92.2 (54.7–181.9)	532.06 (320.61–1036.57)	186 (145.5–344.7)	787.68 (616.02–1474.61)
High-middle	24.8 (14–66.7)	214.76 (120.51–579.03)	69.7 (34.7–110.4)	522.75 (261.31–859.96)
High	4.3 (3.5–5.2)	54.69 (43.12–66.82)	9 (6–12.9)	91.79 (61.91–131.49)
**Regions**				
High-income Asia Pacific	0.3 (0.2–0.5)	17.95 (11.69–27.62)	0.6 (0.5–0.8)	36.75 (27.11–50.8)
Central Asia	0 (0–0)	0 (0–0)	0 (0–0)	0 (0–0)
East Asia	18.2 (14.7–21.6)	148.91 (122.43–176.25)	67.1 (24–126.2)	494.33 (173.26–942.48)
South Asia	180.7 (46.6–464.3)	1663.51 (444.97–4250.09)	312.4 (120.5–765)	1740.79 (660.93–4287.12)
Southeast Asia	48.9 (27.7–129.4)	1036.23 (600.63–2682.51)	77 (70–85.6)	1153.57 (1049.49–1281.59)
Australasia	0.4 (0.1–1)	185.77 (25.62–483.21)	0.6 (0.1–1.4)	188.65 (34.24–466.29)
Caribbean	2.9 (1.9–4)	819.65 (560.26–1141.61)	3.5 (2–5.4)	747.08 (427.62–1140.19)
Central Europe	0 (0–0)	0 (0–0)	0 (0–0)	0 (0–0)
Eastern Europe	0 (0–0)	0 (0–0)	0 (0–0)	0 (0–0)
Western Europe	0 (0–0)	0 (0–0)	0 (0–0)	0 (0–0)
Andean Latin America	1.7 (1–2.6)	455.98 (299.54–665.71)	4 (3.3–4.9)	632.56 (518.08–754.08)
Central Latin America	11.5 (10.5–12.6)	713.32 (633.97–797.16)	19.4 (16.3–22.6)	771.16 (651.56–892.79)
Southern Latin America	0.7 (0.1–1.9)	151.83 (26.41–381.95)	1.1 (0.4–2.5)	167.41 (51.67–376.2)
Tropical Latin America	10.9 (7.3–14.8)	710.56 (481.73–959.89)	22.3 (19.7–24.8)	990.1 (891.27–1089.11)
North Africa and Middle East	3.4 (0.5–7.4)	100.95 (20.62–209.55)	7 (0.9–15)	115.37 (17.71–242.93)
High-income North America	0.1 (0.1–0.2)	4.98 (3.56–6.36)	0.2 (0.2–0.3)	6.44 (4.61–8.32)
Oceania	0.6 (0.5–0.7)	1009.45 (793.94–1198.93)	4.2 (1–8.2)	3173.48 (762.33–6161.18)
Central Sub-Saharan Africa	3.4 (1.6–5.5)	617.81 (313.33–989.16)	6.8 (2–12.7)	539.27 (197.31–961.59)
Eastern Sub-Saharan Africa	8.2 (3.9–12.8)	435.67 (221.9–671.89)	15.7 (4.6–27.8)	382.19 (121.33–666.8)
Southern Sub-Saharan Africa	0.3 (0.2–0.6)	59.7 (33.39–106.06)	0.4 (0.1–0.9)	48.63 (13.05–111.58)
Western Sub-Saharan Africa	14.5 (8.7–20.3)	769.29 (484.42–1055.63)	26.3 (6.3–47.5)	606.75 (201.38–1033.46)

## Data Availability

Data available in a publicly accessible repository. The data presented in this study are openly available in the Global Health Data Exchange GBD Results Tool (http://ghdx.healthdata.org/gbd-results-tool, accessed on 8 August 2022).

## Supplementary Materials

The following are available online at https://www.mdpi.com/article/10.3390/tropicalmed7080180/s1, Figure S1: (a)Age-standardized incidence rate per 100,000 population with dengue of 5 SDI areas in 1990 and 2019; (b) Age-standardized incidence rate per 100,000 population with dengue of 21 regions in 1990 and 2019; (c) Age-standardized incidence rate per 100,000 population with dengue of different genders in 1990 and 2019, Figure S2: Age-standardized DALY rate per 100,000 population with dengue of Oceania countries in 1990 and 2019, Figure S3: Age-standardized incidence rate per 100,000 population with dengue of South Asia countries in 1990 and 2019, Figure S4: Age-standardized DALY rate per 100,000 population with dengue of South Asia countries in 1990 and 2019, Figure S5: Age-standardized incidence rate per 100,000 population with dengue of Southeast Asia countries in 1990 and 2019, Figure S6: Age-standardized DALY rate per 100,000 population with dengue of Southeast Asia countries in 1990 and 2019, Table S1: The Incident cases and age-standardized incidence of Dengue in 1990 and 2019 in Oceania, Table S2: The DALY and age-standardized DALY rate of Dengue in 1990 and 2019 in Oceania, Table S3: The Incident cases and age-standardized incidence of Dengue in 1990 and 2019 in South Asia, Table S4: The DALY and age-standardized DALY rate of Dengue in 1990 and 2019 in South Asia, Table S5: The Incident cases and age-standardized incidence of Dengue in 1990 and 2019 in Southeast Asia, Table S6: The DALY and age-standardized DALY rate of Dengue in 1990 and 2019 in Southeast Asia, Table S7: The age distribution of dengue incidence and DALY in 2019 of Oceania, South Asia and Southeast Asia (Per 100,000).
