# Supplementary material for: Dengue Incidence Trends and Its Burden in Major Endemic Regions from 1990 to 2019
Source: Trop Med Infect Dis. 2022 Aug 12;7(8):180. doi: 10.3390/tropicalmed7080180 (PMC9416661; doi:10.3390/tropicalmed7080180)
Supplement: Supplementary file 1 [file tropicalmed-07-00180-s001.zip › tropicalmed-1833356-supplementary.pdf]

Tropical Medicine and Infectious Disease

MDPI

Supplementary Materials: Dengue Incidence Trends and Its Burden in Major Endemic Regions from 1990 to 2019

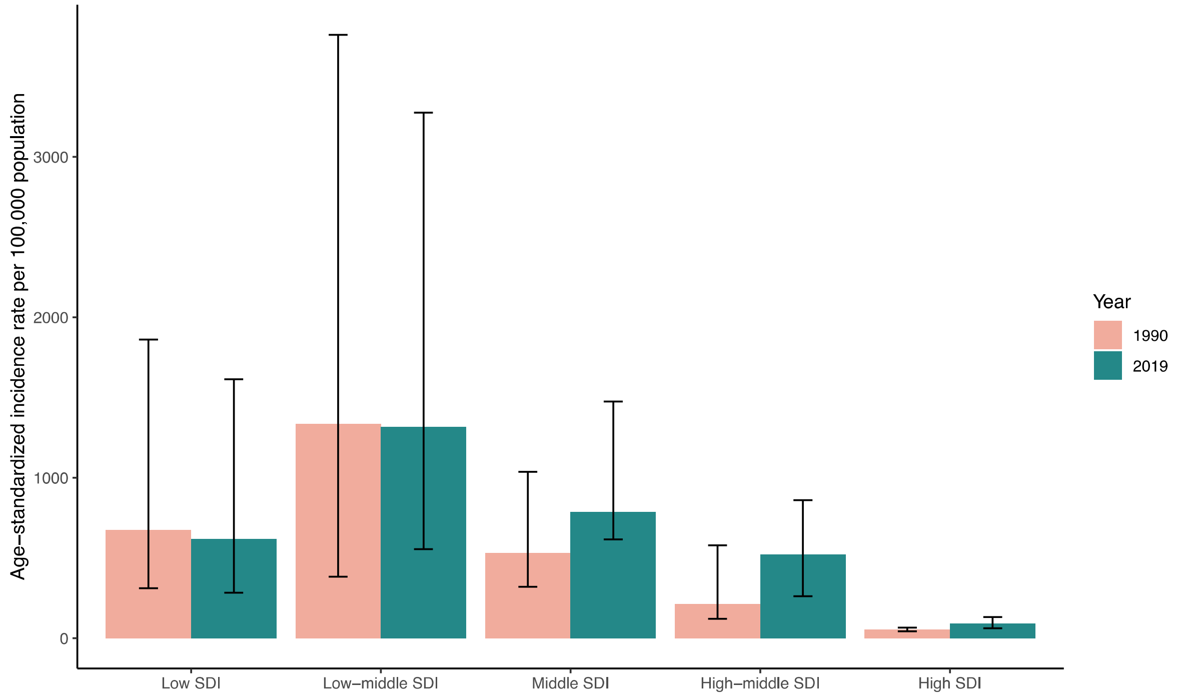

(a)

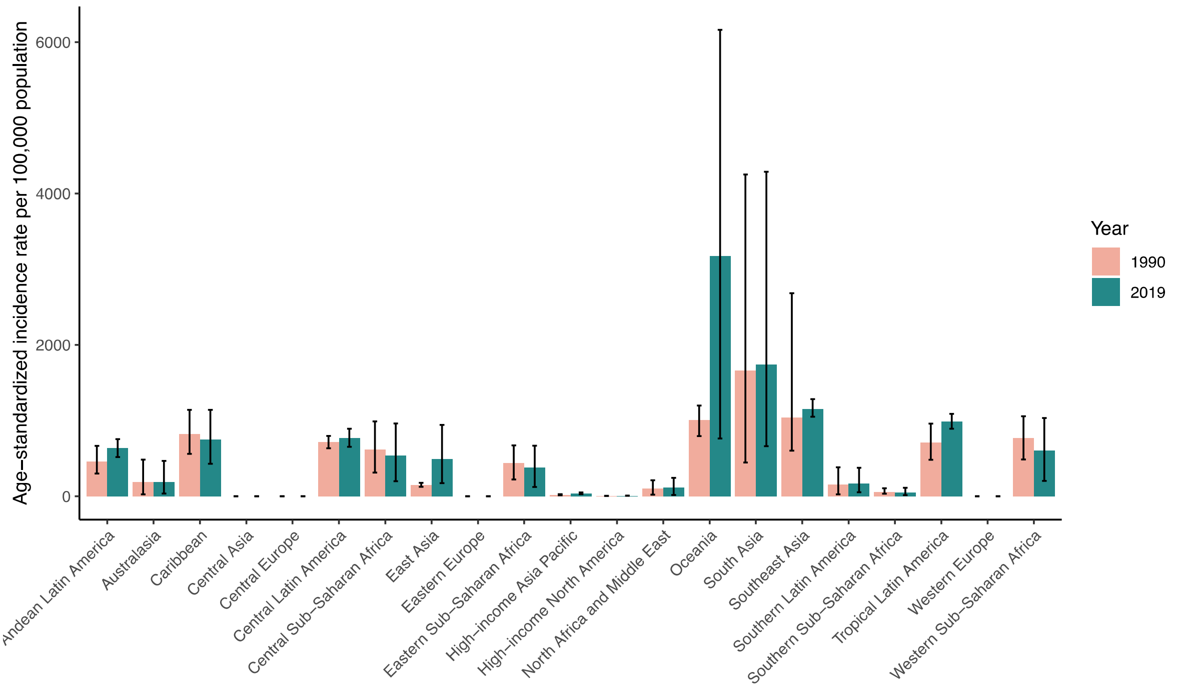

(b)

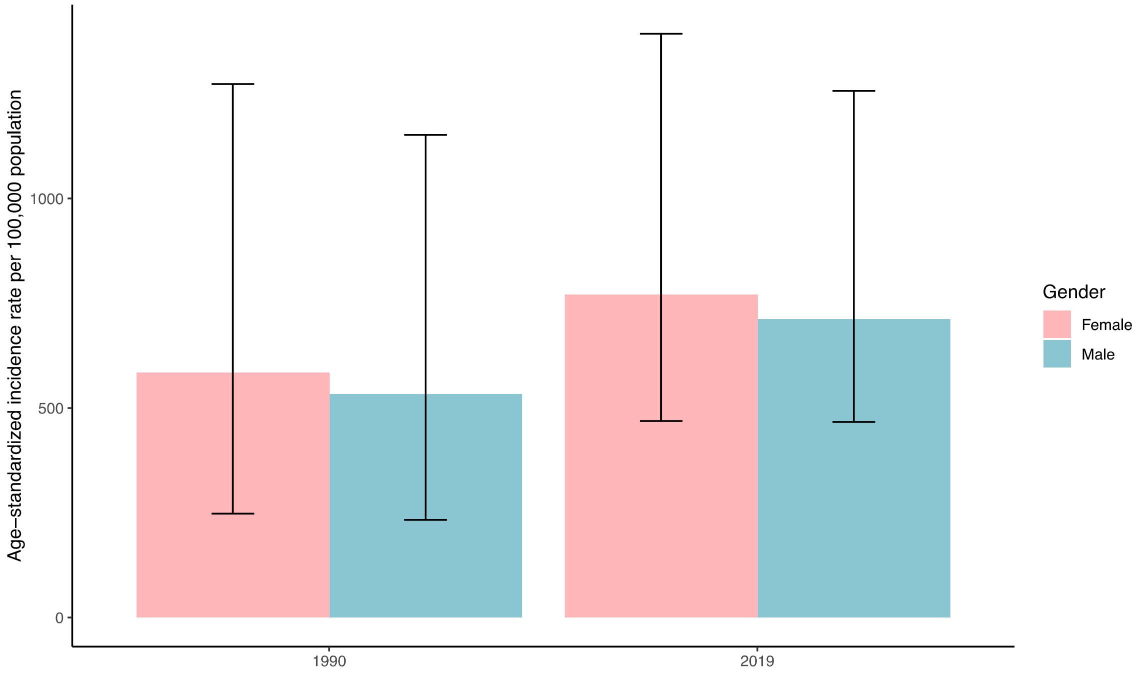

(c)

Figure S1. (**a**). Age-standardized incidence rate per 100,000 population with dengue of 5 SDI areas in 1990 and 2019; (**b**). Age-standardized incidence rate per 100,000 population with dengue of 21 regions in 1990 and 2019; (**c**). Age-standardized incidence rate per 100,000 population with dengue of different genders in 1990 and 2019.

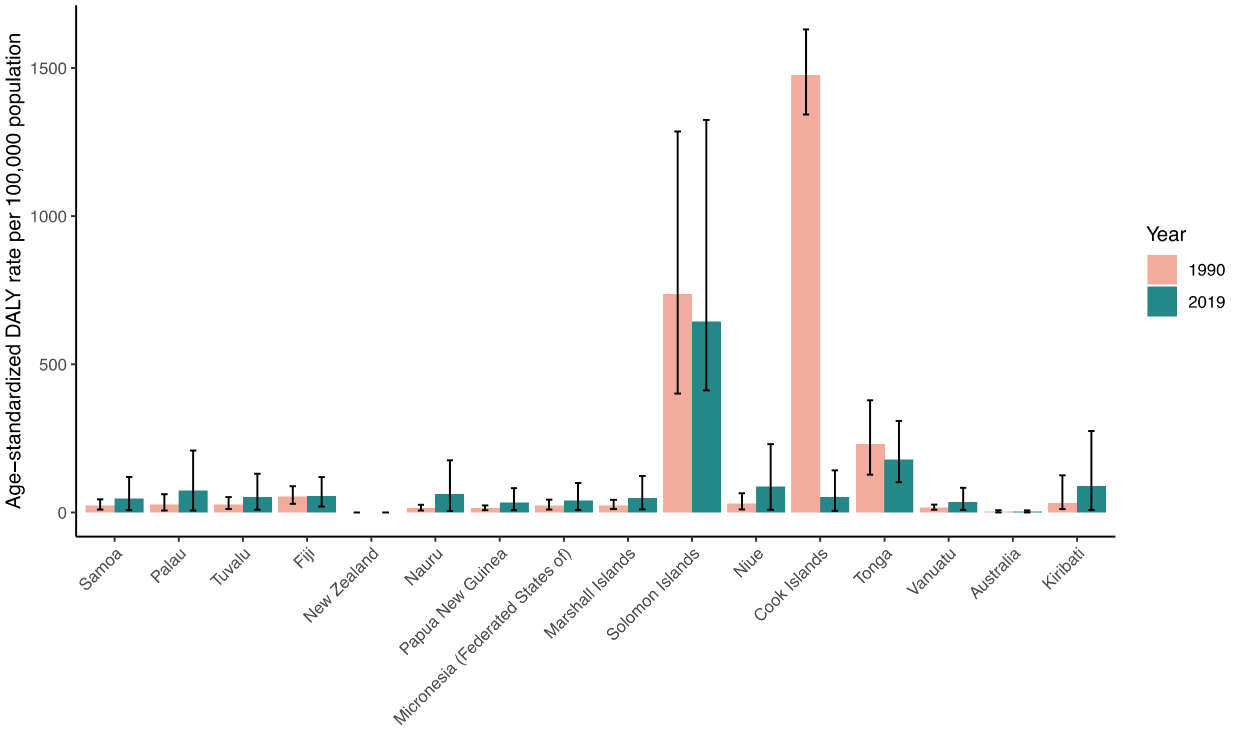

Figure S2. Age-standardized DALY rate per 100,000 population with dengue of Oceania countries in 1990 and 2019.

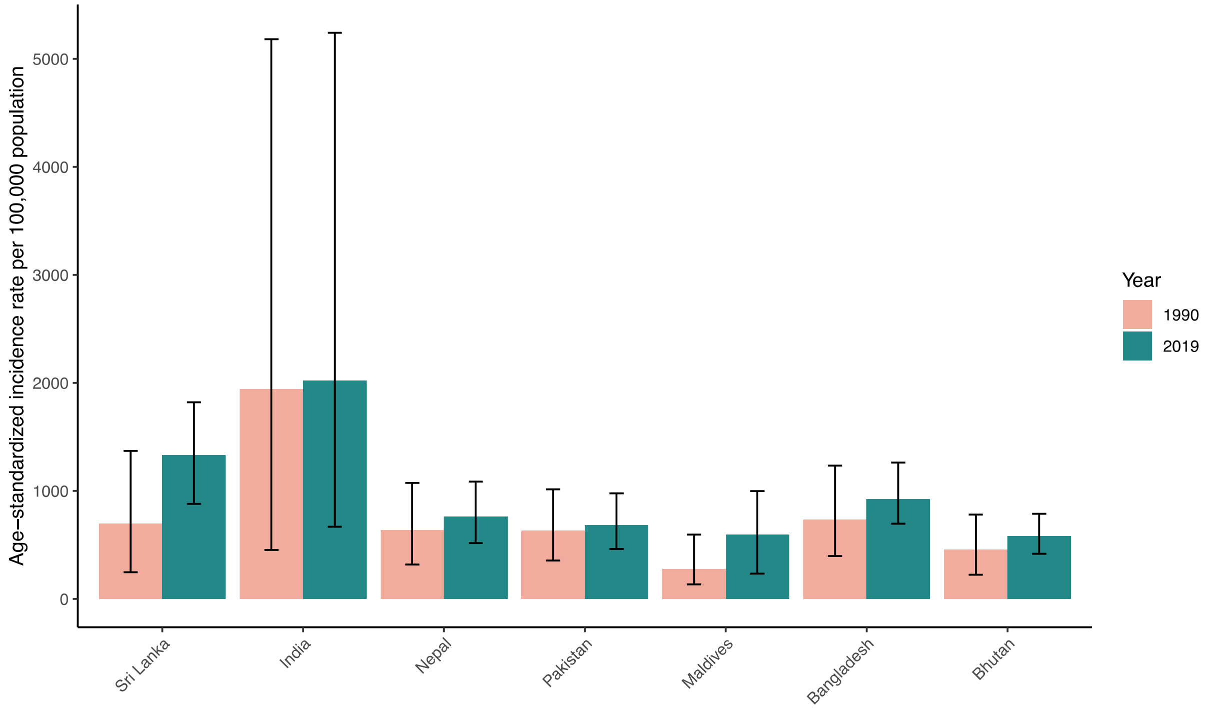

Figure S3. Age-standardized incidence rate per 100,000 population with dengue of South Asia countries in 1990 and 2019.

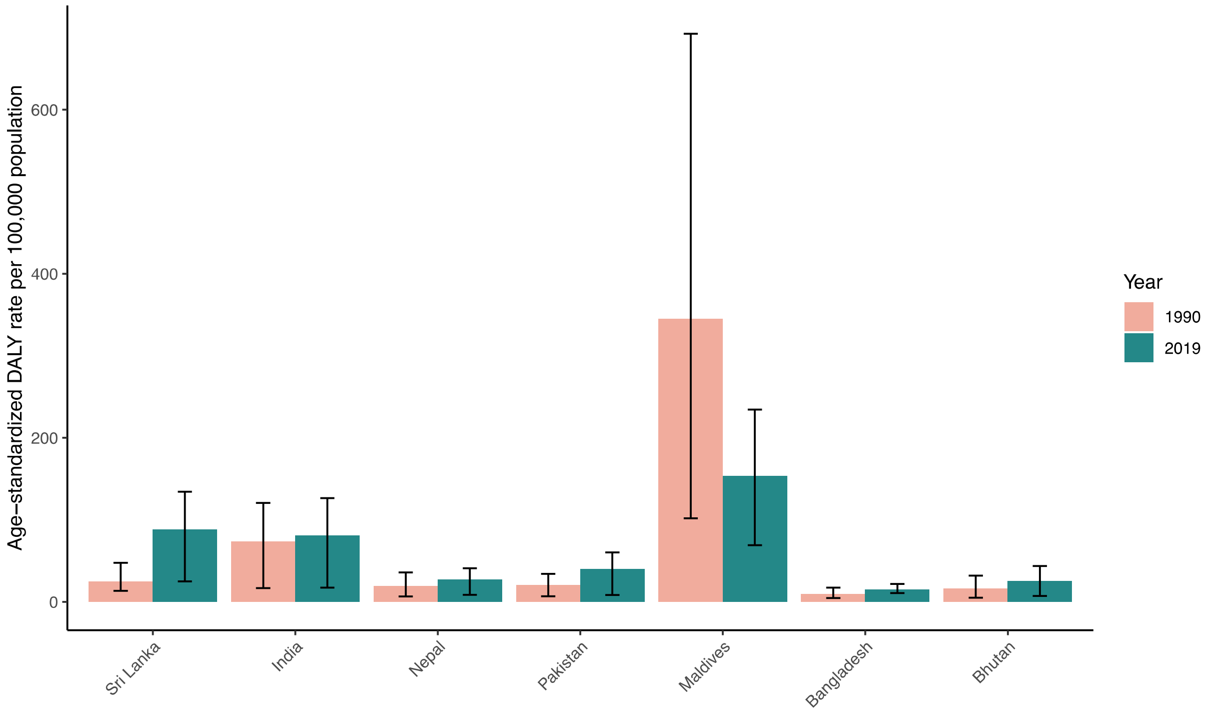

Figure S4. Age-standardized DALY rate per 100,000 population with dengue of South Asia countries in 1990 and 2019.

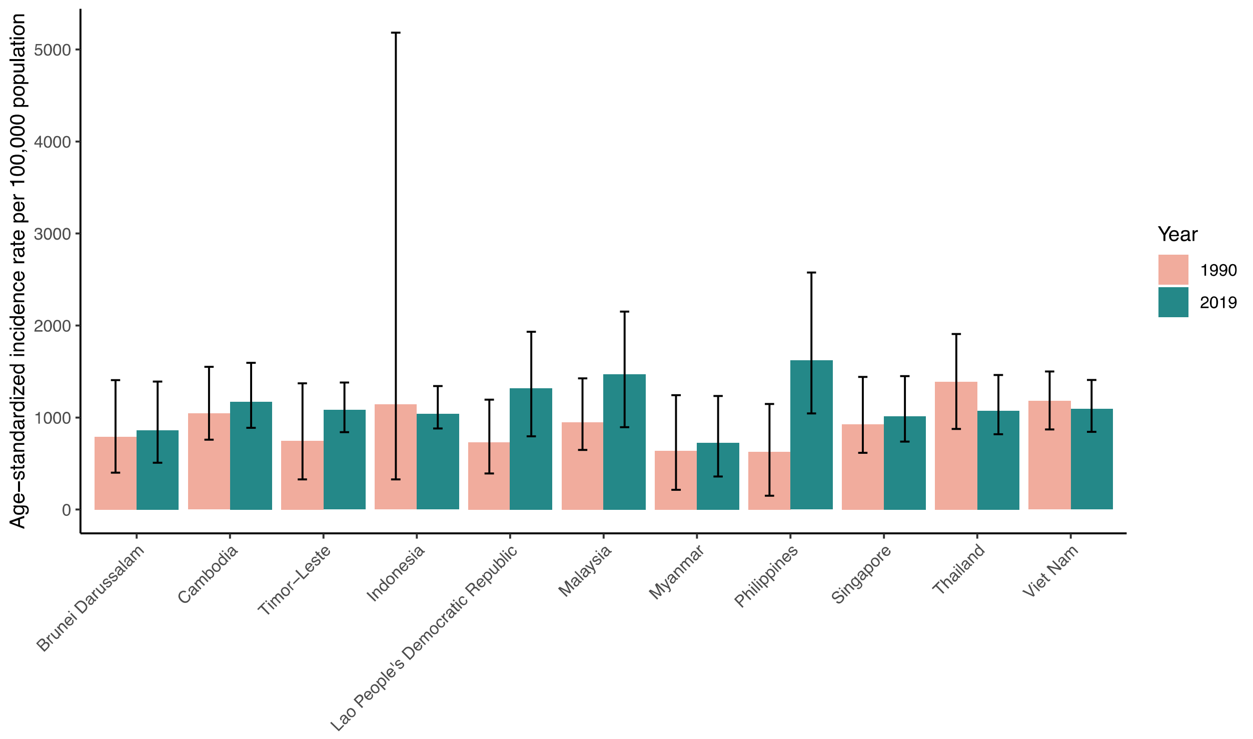

Figure S5. Age-standardized incidence rate per 100,000 population with dengue of Southeast Asia countries in 1990 and 2019.

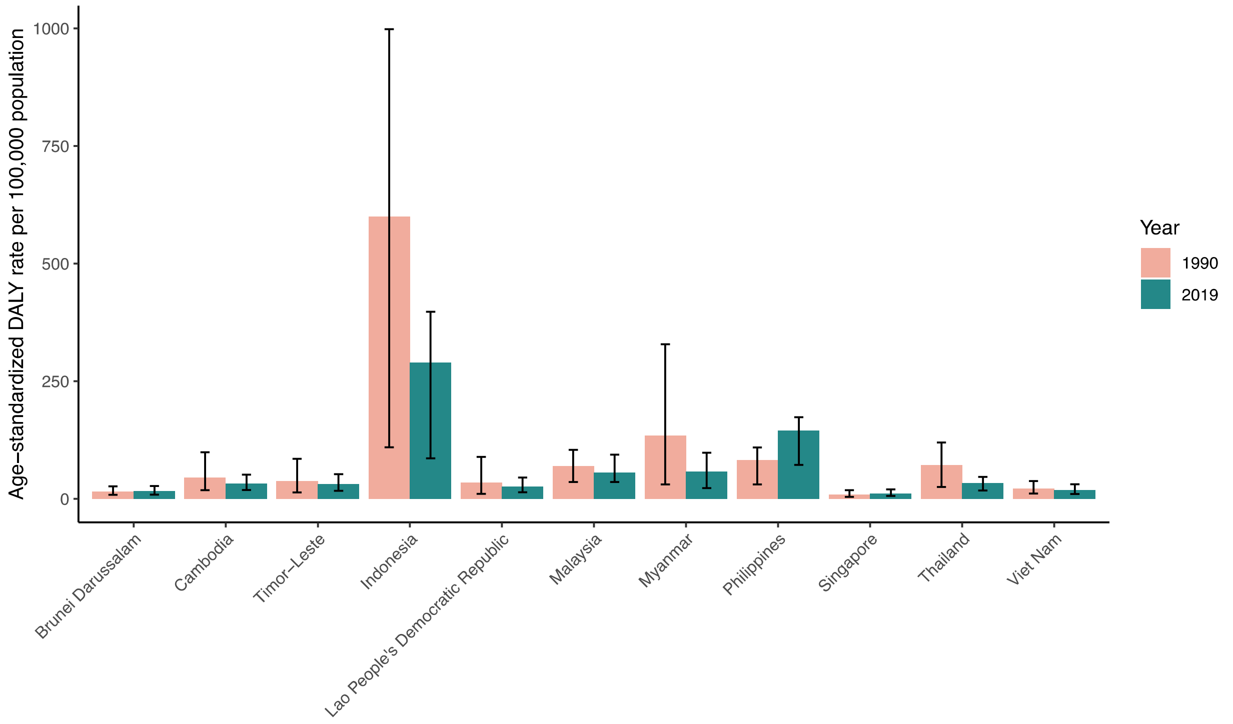

Figure S6. Age-standardized DALY rate per 100,000 population with dengue of Southeast Asia countries in 1990 and 2019.

Table S1. The Incident cases and age-standardized incidence of Dengue in 1990 and 2019 in Oceania.

Characteristics	1990		2019	
	Incident Cases	ASR per 100,000	Incident Cases	ASR per 100,000
	No. X 10^5 (95%UI)	No. (95%UI)	No. X 10^5 (95%UI)	No. (95%UI)
Australia	37610.8 (5340.2–97520.6)	223.62 (30.81–581.71)	55235 (10394.6–136155.6)	223.32 (40.49–552.18)
New Zealand	0(0–0)	0(0–0)	0(0–0)	0(0–0)
Palau	405 (129.5–776.3)	2510.37 (842.14–4696.64)	1305 (155.3–2734.3)	7536.07 (888.63–15922.13)
Micronesia	1701.3 (724.3–3116.7)	1497.1 (686.55–2615.09)	3771 (700.8–7895)	3648.91 (685.84–7429.88)
Marshall Islands	796.5 (399.2–1322.3)	1642.68 (857.84–2568.06)	2520.1 (499–5261.3)	4441 (881.71–9121.69)
Nauru	52 (22.4–84)	491.97 (219.78–767.97)	705.9 (24.4–1632.8)	5703.39 (231.39–13013.35)
Papua New Guinea	24695 (12001–41839.9)	628.76 (347.3–1021.92)	288036.9 (69069.5–590411.7)	2943.6 (705.36–5895.13)
Solomon Islands	3723.8 (2594.8–5576.1)	1047.85 (726.51–1609.95)	25591.1 (5633.4–53449.7)	3797.55 (860.73–7657.75)
Vanuatu	1929.9 (1290.4–2722.2)	1276.67 (849.53–1756.58)	9165.1 (2467.6–17992.3)	3289.71 (833.21–6372.83)
Tuvalu	140.6 (74.8–232.7)	1506.33 (811.8–2473.9)	561.3 (94.3–1171)	4677.83 (797.77–9631.44)
Fiji	15099.2 (6499–26233.3)	2158.6 (852.49–3676.16)	35659.7 (7920.4–71836.2)	3983.59 (878.61–7962.74)
Samoa	3069.4 (1289–5639.3)	1736.76 (770.67–3016.8)	9819.4 (1700.3–20776.7)	4444.48 (792.06–9150.63)
Kiribati	1366.1 (693.1–2223)	1809.97 (939.55–2860.67)	9714.6 (1115.8–21058.1)	8050.59 (944.28–17167.95)
Tonga	3957.2 (807.5–8287.6)	3905.13 (821.13–8019.19)	3284.2 (846.1–6452.8)	3312.81 (819.65–6477.92)
Cook Islands	315.1 (114.3–594.5)	1591.25 (598.52–2946.57)	944.8 (107.8–2001.7)	5246.76 (601.99–11243.99)
Niue	63.9 (21.7–117.5)	2707.12 (920.78–4924.34)	146.5 (16–306.7)	8749.54 (958.61-18365.54)

Table S2. The DALY and age-standardized DALY rate of Dengue in 1990 and 2019 in Oceania.

Characteristics	1990		2019	
	DALY (95% UI)	Per 100,000 Person-Years (95% UI)	DALY (95% UI)	Per 100,000 Person-Years (95% UI)
Australia	380.49 (33.55–1232.99)	2.26 (0.19–7.36)	556.58 (70.46–1690.31)	2.26 (0.27–6.98)
New Zealand	0 (0–0)	0 (0–0)	0 (0–0)	0 (0–0)
Palau	4.02 (0.96–10.00)	25.13 (6.59–61.35)	12.54 (1.15–35.79)	72.87 (6.56–208.75)
Micronesia	24.72 (10.39–49.50)	22.27 (9.79–43.06)	41.53 (8.25–104.99)	40.12 (8.26–99.87)
Marshall Islands	10.91 (5.04–20.97)	23.07 (11.16–42.52)	27.81 (5.77–70.34)	48.81 (10.31–123.34)
Nauru	1.49 (0.69–3.00)	13.42 (6.57–26.06)	7.59 (0.55–21.89)	61.70 (5.16–175.88)

Papua New Guinea	629.47 (305.77–1202.01)	13.84 (7.44–24.10)	3454.94 (817.45–8478.14)	34.01 (8.02–82.29)
Solomon Islands	3048.62 (1450.63–5258.09)	736.51 (400.88–1285.77)	4586.11 (2922.71–8629.23)	643.73 (412.16–1325.22)
Vanuatu	25.03 (14.06–40.77)	16.35 (9.17–26.75)	99.04 (26.69–233.30)	34.91 (8.99–83.06)
Tuvalu	2.59 (1.12–5.24)	26.36 (11.93–51.76)	6.08 (1.11–15.61)	50.66 (9.42–130.70)
Fiji	397.77 (221.02–663.89)	53.17 (28.98–88.27)	490.89 (175.63–1070.99)	54.93 (20.00–119.55)
Samoa	39.12 (15.91–78.43)	22.49 (9.61–44.11)	103.03 (16.62–265.47)	46.63 (8.01–119.97)
Kiribati	24.56 (8.88–100.89)	31.71 (11.49–125.14)	106.68 (10.15–330.60)	88.04 (8.52–275.07)
Tonga	228.29 (121.58–373.13)	231.16 (127.71–378.25)	180.81 (102.78–311.24)	178.12 (101.98–308.74)
Cook Islands	224.49 (204.28–247.92)	1475.96 (1343.00–1630.60)	9.15 (0.96–24.94)	51.22 (5.39–141.93)
Niue	0.72 (0.24–1.54)	30.57 (10.21–64.93)	1.44 (0.15–3.81)	86.70 (9.32–230.66)

Table S3. The Incident cases and age-standardized incidence of Dengue in 1990 and 2019 in South Asia.

Characteristics	1990		2019	
	Incident Cases	ASR per 100,000 No. (95%UI)	Incident Cases	ASR per 100,000 No. (95%UI)
Sri Lanka	120407.66 (42029.91–236749.71)	696.81 (247.67–1370.52)	285258.85 (190816.66–386191.67)	1329.64 (879.91–1821.17)
India	16443576.41 (3726595.02–44042934.66)	1943.39 (453.69–5181.79)	27991970.66 (9433373.98–72604214.80)	2020.83 (667.80–5240.95)
Nepal	122776.33 (56786.79–209781.88)	639.36 (318.77–1075.22)	234986.17 (159286.95–331568.04)	764.25 (517.47–1085.56)
Pakistan	702318.47 (370915.27–1145151.36)	632.98 (356.23–1014.29)	1532033.59 (1018914.43–2188538.23)	684.84 (463.16–977.69)
Maldives	612.89 (284.03–1331.01)	275.46 (135.82–596.06)	3025.12 (1129.22–5315.17)	593.17 (233.42–997.96)
Bangladesh	794158.01 (404701.24–1358937.09)	734.57 (396.90–1235.26)	1481551.66 (1118976.10–2011114.36)	922.89 (697.19–1262.38)
Bhutan	2792.11 (1269.32–4819.88)	457.70 (223.91–781.44)	4413.27 (3154.16–5928.35)	580.95 (417.56–789.63)

Table S4. The DALY and age-standardized DALY rate of Dengue in 1990 and 2019 in South Asia.

Characteristics	1990		2019	
	DALY (95% UI)	Per 100,000 person-years (95% UI)	DALY (95% UI)	Per 100,000 person-years (95% UI)
Sri Lanka	4341.66	19.31	7746.26	27.17

	(1296.94–9090.58)	(6.66–35.96)	(2516.77–11963.86)	(8.54–40.92)
India	109.59 (27.28–244.54)	16.24 (4.94–32.10)	175.41 (51.99–306.55)	25.36 (7.24–43.66)
Nepal	656354.76 (142644.92–1096380.18)	73.33 (16.80–120.49)	1066687.41 (230921.41–1686113.73)	80.63 (17.47–126.48)
Pakistan	26485.06 (7732.77–50064.60)	20.25 (6.78–34.14)	91032.36 (18826.64–145056.16)	39.67 (8.44–60.32)
Maldives	10274.71 (4820.55–19557.21)	9.35 (4.56–17.34)	23680.64 (16359.38–34198.24)	15.29 (10.70–21.82)
Bangladesh	4420.07 (2429.18–8397.55)	24.46 (13.48–47.47)	18138.32 (5145.68–27420.50)	87.78 (24.89–134.18)
Bhutan	1103.45 (273.02–2388.75)	345.10 (101.88–692.54)	736.40 (330.77–1141.39)	153.32 (69.16–234.50)

Table S5. The Incident cases and age-standardized incidence of Dengue in 1990 and 2019 in Southeast Asia.

**Characteristics**	**1990**		**2019**	
	**Incident Cases No. (95%UI)**	**ASR per 100,000 No. (95%UI)**	**Incident Cases No. (95%UI)**	**ASR per 100,000 No. (95%UI)**
Brunei Darussalam	2019.8 (982.6–3608.1)	792.25 (401.83–1405.85)	3828.6 (2402.9–5941)	861.24 (507.74–1391.93)
Cambodia	107133.5 (73860.5–160987.5)	1043.82 (758.14–1551.06)	194685.4 (147095.6–263078.9)	1171.37 (887.8–1594.67)
Timor-Leste	5738.6 (2346.1–10647)	748.17 (327.61–1371.13)	14697.3 (11236.2–19087.7)	1081.21 (840.7–1380.49)
Indonesia	2173180.7 (618623–9995522.3)	1143.41 (328.46–5182.99)	2655179.6 (2243011.3–3450631.1)	1037.02 (881.07–1342.97)
Lao People's Democratic Republic	28388.2 (12698.2–49709.1)	728.86 (392.69–1193.72)	94474.4 (56762.8–140588.7)	1319.73 (796.44–193.54)
Malaysia	167407.3 (111590.5–252175.5)	947.42 (648.65–1425.78)	457683.9 (277434.7–675960.6)	1468.76 (894.29–2150.69)
Myanmar	263713.3 (87015.5–512501.4)	639.4 (215.06–1243.97)	392553.7 (192026.1–669275.6)	724.6 (358.65–1233.58)
Philippines	392056.6 (79369.6–730089.9)	629.16 (148.95–1147.73)	1832711.7 (1165380.1–2979228.3)	1619.95 (1044.81–2574.39)
Singapore	28728.5 (19511.8–43441.2)	928.3 (615.9–1441.33)	58228.5 (43929.1–79529.3)	1013.87 (738.37–1448.53)
Thailand	798026.4 (499380.4–1113112.3)	1389.11 (875.56–1906.71)	699667.8 (508746.2–1009396.9)	1071.76 (817.13–1461)
Vietnam	812956.1 (598469.7–1051770.6)	1179.1 (870.85–1500.71)	1038967.7 (812230.9–1333875)	1090.57 (843.72–1408.31)

Table S6. The DALY and age-standardized DALY rate of Dengue in 1990 and 2019 in Southeast Asia.

Characteristics	1990		2019	
	DALY (95% UI)	Per 100,000 person-years (95% UI)	DALY (95% UI)	Per 100,000 person-years (95% UI)
Brunei Darussalam	37.73 (19.79–65.03)	15.75 (8.52–26.41)	74.14 (40.77–119.53)	16.65 (8.87–27.45)
Cambodia	6577.63 (2234.63–15435.98)	44.97 (18.30–99.05)	5484.80 (3214.38–8972.49)	31.91 (18.90–51.33)
Timor-Leste	431.01 (123.22–1098.54)	37.50 (13.45–85.03)	472.88 (250.76–837.11)	30.82 (17.16–52.37)
Indonesia	1322558.24 (227422.46–2223416.07)	600.02 (109.48–998.23)	647505.72 (205032.78–879508.99)	290.10 (85.96–397.48)
Lao People's Democratic Republic	1994.24 (458.05–5580.21)	35.07 (10.48–88.97)	1965.84 (1031.27–3403.61)	26.20 (13.91–45.23)
Malaysia	13363.08 (7069.55–20556.98)	70.32 (35.78–104.04)	17625.25 (11187.02–29021.27)	55.95 (35.96–93.76)
Myanmar	73348.89 (15076.86–187701.77)	134.12 (30.52–328.49)	30012.74 (11879.34–50279.95)	57.88 (22.55–97.73)
Philippines	69048.47 (22401.86–92265.10)	82.73 (30.62–109.37)	177024.63 (86148.37–212202.78)	145.51 (72.15–173.75)
Singapore	283.62 (131.11–558.35)	9.13 (4.11–18.64)	684.39 (385.23–1143.49)	11.45 (6.22–20.03)
Thailand	41831.99 (14808.59–68997.52)	72.39 (25.33–119.45)	18978.36 (10420.42–26957.35)	33.75 (17.72–46.73)
Vietnam	16311.85 (8309.75–29605.24)	21.50 (11.15–37.83)	16786.91 (9661.52–27316.66)	18.70 (10.40–30.98)

Table S7. The age distribution of dengue incidence and DALY in 2019 of Oceania, South Asia and Southeast Asia (Per 100 000).

Age groups	Oceania		South Asia		Southeast Asia	
	Age-standardized incidence rate No. (95%UI)	DALY person-years (95% UI)	Age-standardized incidence rate No. (95%UI)	DALY person-years (95% UI)	Age-standardized incidence rate No. (95%UI)	DALY person-years (95% UI)
**Under 5 years**	1622.76 (540.73–4000.98)	134.77 (64.16–222.86)	1033.22 (253.93–2720.77)	156.11 (21.36–173.13)	703.54 (479.67–875.47)	726.63 (176.49–1061.41)
**5–14 years**	3916.97	60.19	1927.67	58.05	1447.19	165.86

	(930.97–8969.21)	(22.05–128.49)	(642.01–4783.93)	(14.36–102.97)	(1263.18–1823.01)	(66.35–209.75)
**15–49 years**	3241.41 (746.59–6719.03)	66.36 (29.66–148.18)	1746.37 (727.36–4170.48)	54.99 (12.59–86.55)	1162.85 (1013.91–1370.47)	72.86 (36.42–96.06)
**50–69 years**	2694.36 (702.33–5665.02)	48.84 (21.23–102.92)	1697.99 (581.51–4209.18)	72.05 (13.62–106.53)	935.82 (757.16–1124.07)	59.38 (22.06–76.73)
**Over 70 years**	4610.21 (879.78–10138.58)	66.78 (23.62–156.11)	2284.13 (683.85–5983.69)	137.06 (38.12–193.18)	1356.32 (1222.49–1538.18)	66.79 (33.89–83.03)
